# Progress of lymphocyte activation gene 3 and programmed cell death protein 1 antibodies for cancer treatment: A review

**DOI:** 10.17305/bb.2024.10339

**Published:** 2024-08-01

**Authors:** Yu-Quan Li, Xue-Mei Chen, Gui-Fei Si, Xue-Min Yuan

**Affiliations:** 1 School of Clinical Medicine, Shandong Second Medical University, Weifang, China; 2 Linyi People’s Hospital, Linyi, China

**Keywords:** Immunotherapy, programmed cell death protein 1 (PD-1), programmed cell death protein 1 ligand 1 (PD-L1), lymphocyte activation gene 3 (LAG-3), combination therapy

## Abstract

The application of immune checkpoint inhibitors (ICIs) has proven to be an effective treatment for cancer. Immune checkpoints, such as programmed cell death protein 1/programmed cell death protein 1 ligand 1 (PD-1/PD-L1), cytotoxic T-lymphocyte-associated protein 4 (CTLA-4), T-cell immunoglobulin-3 (TIM-3), T-cell immunoglobulin and ITIM domain (TIGIT), and lymphocyte activation gene 3 (LAG-3) have received extensive attention, and the efficacy of antibodies or inhibitors against these checkpoints (either alone or in combination) has been evaluated in many tumors. This paper provides a brief overview of the PD-1 and LAG-3 checkpoints and then shifts focus to the combined use of PD-1 and LAG-3 antibodies in both in vivo and in vitro experiments. In the in vitro experiments, we examined the correlation between the expression and activation of these inhibitors on T cells, and also assessed toxicity in animals in preparation for in vivo experiments. The effects of the combined use of PD-1 and LAG-3 antibodies were then summarized in animal models of melanoma, MC38 carcinoma, and other tumors. In clinical studies, the combined application of these antibodies was assessed in patients with melanoma, colorectal, breast, and renal cell cancers, as well as other solid tumors. In general, the combination of PD-1 and LAG-3 antibodies has shown promising results in both in vivo and in vitro studies.

## Introduction

Treating cancer has long been a challenging task for humans. Advances in surgeries, radiotherapy, and chemotherapy have led to improved outcomes for many cancer patients. However, those with advanced cancer continue to face a poor prognosis. As our understanding of the immune system has deepened, researchers have explored using immune cells to target and eliminate cancer. This has sparked significant interest in tumor immunotherapy [1]. One of the key research areas is immune checkpoints, such as programmed cell death protein 1 (PD-1), programmed cell death protein 1 ligand 1 (PD-L1), cytotoxic T-lymphocyte-associated protein 4 (CTLA-4), lymphocyte activation gene-3 (LAG-3), T-cell immunoglobulin-3 (TIM-3), and T-cell immunoglobulin and ITIM domain (TIGIT) [2]. After a large number of studies and trials were conducted, antibodies against these immune checkpoints have been progressively developed, such as nivolumab, pembrolizumab, and ipilimumab, among other antibodies [1, 3], and these antibodies have achieved good results. However, there are still a considerable number of patients with a low response rate or serious adverse events (AEs) [1].

**Figure 1.**
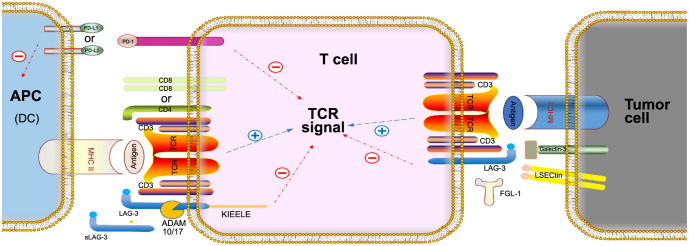
**Immunomodulatory effects of LAG-3 and PD-1.** After LAG-3 binds to MHC class II, FGL-1, LSECtin, and galectin-3, or when PD-1 binds to PD-L1/L2, inhibitory signals are transmitted to T cells, thus leading to the inhibition of effector T-cell function and T-cell exhaustion. FGL-1: Fibrinogen-like protein 1; LSECtin: Liver sinusoidal endothelial cell lectin; sLAG-3: The soluble form of LAG-3; MHC II: Major histocompatibility complex class II; APC: Antigen presenting cell; TCR: T-cell receptor; LAG-3: Lymphocyte activation gene 3; PD-1: Programmed cell death protein 1.

To enhance treatment efficacy, researchers have introduced immune-based combination therapy, which involves combining immunotherapy with chemotherapy or tyrosine kinase inhibitors (TKIs) [4]. For example, some researchers treat renal cell carcinoma (RCC) with immune checkpoint inhibitors (ICIs) plus TKIs [5], while breast cancer is treated with ICIs plus ladiratuzumab (targeting LIV-1) [6]. Moreover, immune-based combinations or ICI monotherapy are being explored as adjuvant treatment for hepatocellular carcinoma (HCC) [7]. However, immune-based combination therapy is also thought to be associated with some AE, especially hypertransaminasemia [8]. Another option is to combine two different ICIs. Among them, the most concerning treatments are the combination of PD-1 and CTLA-4, and PD-1 and LAG-3 [1, 9]. The combined application of PD-1 and CTLA-4 antibodies was initiated at an early stage and has been widely used in clinical practice with a profound lasting response rate and controllable AEs, which has significantly changed the treatment of advanced cancer [1]. However, there are still many patients who cannot benefit from this treatment; thus, clinical personnel have begun to utilize other combination treatments, such as PD-1/PD-L1 and LAG-3 [10]. The combination of PD-1/PD-L1 and LAG-3 has been applied by many scholars in the clinical setting, including applications with many types of tumors. This paper analyzes and discusses the combined application of PD-1/PD-L1 and LAG-3. First, this review introduces the basic function of PD-1 and LAG-3; additionally, when considering the fact that there is much literature in this field, this paper will provide a brief summary of these concepts and subsequently focus on the preclinical and clinical applications of the combination of PD-1/PD-L1 and LAG-3.

### Programmed cell death protein 1 (PD-1)

PD-1 (PDCD1 and CD279) is a common cell surface receptor found in B cells, T cells, and natural killer (NK) cells [1]. Studies on PD-1 have mainly focused on T cells, with less research being conducted on B cells or NK cells. It is a transiently expressed gene that was discovered by Professor Tasuko Honjo and his colleagues [11]. Among other emerging negative regulatory receptors that mediate these inhibitory feedbacks, PD-1 has become one of the most studied regulatory factors, due to its indispensable role in fine-tuning T-cell function and maintaining the dynamic balance of the immune system [12]. The ligands for PD-1, such as PD-L1 and PD-L2, are commonly expressed in dendritic cells (DCs) and macrophages [13, 14]. Specifically, PD-L1 is expressed on B cells, DCs, macrophages, cultured bone-marrow-derived mast cells, T cells, and non-hematopoietic cell types. In contrast, PD-L2 is inducibly expressed only on DCs, macrophages, and bone-marrow-derived cultured mast cells [15]. When PD-1 binds to its ligands, it inhibits the cell proliferation, cytokine secretion, and cytotoxicity of immune cells, thus weakening the immune response (Figure 1) [16]. After PD-1 activation, SHP1 and SHP2 phosphatases, which inhibit ZAP70 and PI3K activity, are recruited and then downstream ERK and PKCθ intracellular pathways are also terminated. It can also decrease CK2 expression and activity through the PI3K-dependent signaling pathway, resulting in the elimination of PIP3 by active PTEN and thus shut off AKT activation. These will inhibit the activity of T cells [17–20]. Some researchers investigated that ligation of PD-L1 or PD-L2 can lead to reverse signaling into the DC that ultimately results in the inhibition of the ensuing immune response, as PD-L1 and PD-L2 might bidirectionally regulate DC–T cell interactions [15, 21]. In addition to being expressed by conventional T cells, PD-1 is also expressed by some myeloid cell populations and tumor cells, in which we have limited knowledge of its role. In recent years, researchers investigated that anti-PD-1 might regulate myeloid response for antitumor immunity involving a shift in myeloid cell fate away from immature myeloid-derived suppressor cells (MDSCs) and toward differentiated monocytes, macrophages, and DCs [22]. And in tumor cells, the coordination of PD-1 and PD-L1 activates its major downstream signaling pathways including the AKT and ERK1/2 pathways, thus enhancing tumor cell growth [23]. In addition, sPD-1 and sPD-L1 are the soluble counterparts of PD-1 and PD-L1, and studies have shown that sPD-1 could bind PD-L1 and PD-L2 to block PD-1/PD-L1 interaction [24]. Several monoclonal antibodies targeting PD-1 (pembrolizumab, nivolumab, and cemiplimab) or PD-L1 (durvalumab, atezolizumab, and avelumab) for the treatment of hematological and solid malignancies have been approved by the Food and Drug Administration (FDA) [3], including treatments for metastatic melanoma, RCC, metastatic nonsmall cell lung cancer (NSCLC), classical Hodgkin’s lymphoma, metastatic urothelial carcinoma, and HCC [1]. The current research is mainly on PD-1/PD-L1 antibodies, however, PD-L2 is also an advanced candidate. Though some studies have found a greater effect of anti-PD-L1 blockade compared with anti-PD-L2 blockade, more research is also needed to assess anti-PD-L2 blockade value [21].

In addition, PD-L1 is a biomarker of response to immune-checkpoint inhibitors. In most scenarios, only 20%–40% of patients will respond to anti-PD-1/PD-L1 [25]. Multiple studies across many cancers have provided solid evidence about a positive correlation between PD-L1 expression and response to immunotherapy, so PD-L1 can be the biomarker to identify groups of patients who will benefit from these agents [25, 26]. The FDA-approved PD-L1 assays are classified as either “companion” or “complementary” diagnostics [25]. PD-L1 expression can be detected using immunohistochemistry (IHC) [27], however, due to the unique IHC assays and interpretations for each ICI and various preanalytical issues common for all IHC assays, PD-L1 testing and interpretation are not easy to perform [26].

### Lymphocyte activation gene 3 (LAG-3)

LAG-3 (CD223) is a molecule that is upregulated on activated CD4+ and CD8+ T cells and a subset of NK cells and was initially discovered by Triebel et al. in 1990 [28]. The LAG-3 gene is located near CD4 on chromosome 12 in humans [29]. Early studies suggested that LAG-3 defined a specific mode of natural killing on NK cells [30, 31]. Following T-cell receptor (TCR) stimulation, LAG-3 (which is stored in lysosomal compartments) translocates to the cell surface to control T-cell responses [32, 33], and it is also regulated by proteolytic cleavage, thus leading to the shedding of a soluble form of LAG-3 (sLAG-3) [34, 35]. Some researchers proposed that sLAG-3 may function similarly to a synthetic LAG-3 fusion protein (sLAG-3-Ig) to bind to MHCII, thus inhibiting the binding of LAG-3 and its inhibitory function [36, 37]. LAG-3 structurally resembles the CD4 coreceptor but binds to MHC class II with a higher affinity [38]. Moreover, LAG-3 ligands include MHC class II, alternative ligands, and other ligands, such as galectin-3 (Gal-3) and fibrinogen-like protein 1 (FGL-1) [29, 35]. When LAG-3 binds to its ligands, it negatively regulates the activation, proliferation, homeostasis, and effector functions of CD4+ and CD8+ T cells (Figure 1) [39]. One putative mechanism of action is that LAG-3 colocalization with the immune synapse exerts its function [36]. And LAG-3 mainly negatively regulates T-cell activation in three ways. First, negative regulation directly inhibits the activation and proliferation of T cells. Second, the T-cell immune response is suppressed by indirectly promoting the inhibitory function of regulatory T cells. Third, T-cell activation is prevented by regulating antigen-presenting cells (APCs) [39]. LAG-3 expresses also on plasmacytoid DCs (pDCs), LAG-3+ pDC represents 6% of total circulating pDCs, and Lag-3 is a negative regulator of pDC activation [40, 41]. There are a large number of LAG-3-targeted drugs undergoing clinical trials, such as relatlimab, eftilamidol alpha, LAG525, BI754111, TSR-033, and REGN3767, including treatments for melanoma, mesothelioma, breast cancer, lymphoma, myeloma, and leukemia, among other cancers [42, 43]. Current LAG-3-targeted therapies can be categorized into three subtypes: anti-LAG-3 monoclonal antibodies (relatlimab, Sym022, IMP701, MK-4280, and TSR-033), LAG-3-immunoglobulin (Ig) fusion proteins (IMP321), and LAG-3 bispecifics (IBI323, FS118, EMB-02, and MGD013) [43].

### PD-1 and LAG-3 expression correlation analysis and effect testing

There are a considerable number of preclinical studies demonstrating the possibility of this combination regimen. The striking synergy between PD-1 and LAG-3 has been observed in multiple settings [29].

A previous study investigating small cell lung cancer (SCLC) suggests that LAG-3 expression was markedly associated with PD-1 and PD-L1 expression (both *P* < 0.05) with 81 clinical SCLC samples [44]. The combination with PD-1 blockade demonstrated promising results, as immunotherapy with antibody-mediated blockade of LAG-3 alone shows limited efficacy in models of chronic viral infection and cancer, and the dual blockade of PD-1/LAG-3 synergistically reduced viral load by countering CD8+ T-cell exhaustion in chronic lymphocytic choriomeningitis virus infection, thus improving antiviral CD8+ T-cell responses [45]. One study described the binding properties of an anti-human PD-1 antibody and an anti-human LAG-3 antibody [46]. In this vitro model of antigen-experienced memory T cells expressing PD-1 and LAG-3, IFN-γ secretion was increased on average by 13.2 times vs isotype control (*P* < 0.0001) with BI754111 (anti-LAG-3) plus ezabenlimab (anti-PD-1), which was significantly more than BI754111 or ezabenlimab monotherapy, thus supporting the clinical investigation of this combination (NCT03156114; NCT03433898) [46]. In an in vitro functional analysis of allogeneic T cells, the combination of REGN3767 (anti-LAG-3) with cemiplimab (REGN2810, anti-PD-1) increased T-cell activation, the proportion of effector T cells in the tumor and intratumoral CD4+ and CD8+ T cells producing IFN-γ, TNF-α, and IL-10 levels in the blood and spleen to reduce tumor growth [47]. In addition, LAG-3 is regarded as a marker found in PD-1-resistant patients, and anti-LAG-3 antibodies improved antitumor activity in these patients [48–50].

### Toxicities and safety

Immunotherapy can form immune memory, and some patients can achieve long-term remission. But there are AEs that can manifest as autoimmune phenomena and affect any organ, such as arthritis, colitis, hepatitis, or endocrine diseases. And the AEs of different ICIs are somewhat different [51].

The side effects and immune-related AEs (IrAEs) associated with PD-1 blockade mainly include interstitial pneumonitis, colitis with gastrointestinal perforation, type 1 diabetes, severe skin reactions, and immune thrombocytopenia, but they are generally considered to be well tolerated and manageable compared with the toxicity profile of CTLA-4 inhibitors and chemotherapy [12, 52].

When anti-PD-1 antibody (nivolumab) and anti-CTLA4 antibody (ipilimumab) are used together to treat cancer, the combination induced a high response rate with deeper responses than either antibody alone; at the same time, there were higher rates of immune-related toxicities than would be expected with either agent alone [53, 54]. The combination of anti-LAG3 and anti-PD-1 also had a higher rate of treatment-related toxicities than nivolumab alone, but the difference in toxicities between combination therapy and anti-PD-1 monotherapy appeared to be smaller than that between combined anti-PD-1 and anti-CTLA4 and monotherapy [53, 55].

The safety of anti-PD-1/LAG-3 combination has been assessed and demonstrated that 8 of 9 cynomolgus monkeys were generally well tolerated with no adverse clinical symptoms when coadministered relatlimab at 100 mg/kg and nivolumab at 50 mg/kg in a preclinical toxicity evaluation, but one male monkey died due to central nervous system (CNS) vasculitis [56]. Moreover, ABL501, which is a bispecific antibody targeting LAG-3 and PD-L1, effectively enhanced the activation of effector CD4+ and CD8+ T cells to a greater extent than a combination of single anti-LAG-3 and anti-PD-L1; in addition, the safety of ABL501 was also assessed and was well tolerated in cynomolgus monkeys [57].

## Preclinical evidence

### Melanoma and mesothelioma

In a sensitive in vitro model based on expanded autologous tumor-infiltrating lymphocytes (TILs) and melanoma cell lines obtained from tumor specimens of melanoma patients, LAG-3 and PD-1+LAG-3 inhibition promoted antitumor immune responses in human autologous melanoma/T-cell cocultures [58]. Researchers developed a mouse melanoma model in which the initial regression of advanced disease was followed by tumor recurrence, and the combination blockade of the inhibitory molecules PD-L1 and LAG-3 effectively treated recurrent melanoma [59].

In a mesothelioma mouse model, Marcq et al. [60] found that monotherapy with an immune checkpoint blocking antibody against PD-1 and its combination with another blocking antibody against LAG-3 resulted in delayed tumor growth and survival benefits in experimental mice.

### MC38 carcinoma or Sa1N fibrosarcoma, other colon cancers, and ovarian cancer

In Turnis’ study, over 75% of mice with MC38 cancer or Sa1N fibrosarcoma were effectively treated with a combination of anti-PD-1 and anti-LAG-3 antibodies, resulting in complete clearance of tumors and prolonged survival [61]. Another study utilized three different mouse colon cancer cell lines: MC38, MC38.OVA (engineered to express ovalbumin), and CT26. Treatment with the anti-LAG-3/PD-L1 mAb^2^ (bispecific antibody) eliminated tumors in six of eight mice and slowed tumor growth in the remaining two mice [62]. Furthermore, CB213 is a novel asymmetric bispecific antibody that blocks signaling through LAG-3 and PD-1 and inhibits tumor growth in MC38 models [63]. In a prophylactic MC38 ovarian tumor model treated with cemiplimab (anti-PD-1) and REGN3767 (anti-LAG-3), more mice were tumor-free, and tumor growth was significantly reduced on day 22, in the combination treatment group compared with the same-type control group (*P* < 0.05) and the REGN3767 group (*P* < 0.01) [47].

In addition, the anti-LAG-3/PD-L1 mAb^2^ was also used to reduce tumor burden in the MC38 colon cancer model, and there were more tumor-free animals in the LAG-3/PD-L1 bispecific group than in the combined anti-LAG-3 and anti-PD-L1 groups, which was similar to the CT26 mouse colon cancer model [64]. In the ovarian cancer model, the results suggested that the inhibition of the PD-1 or LAG-3 pathways alone was insufficient to control ovarian cancer, whereas the combined blockade with anti-LAG-3 and anti-PD-1 antibodies significantly delayed the growth of IE9mp1 ovarian tumors (*P* ═ 0.01) [65].

### Non-small cell lung cancer (NSCLC), breast cancer, chronic lymphocytic leukemia (CLL), glioblastoma, pancreatic cancer, and prostate cancer

In a previous study, a humanized mouse model of NSCLC was established, which was administered twice weekly with the combination of mouse anti-PD-1 (TSR-042) and anti-LAG-3 (TSR-033); additionally, when tumor growth was monitored for 35 days, this model showed significant synergy and the elimination of tumor growth in most implanted mice (tumor growth inhibition [TGI] effect was 97%; coefficient of drug interaction [CDI] < 0.7), as well as significant increases in proliferating T cells and total CD8+ T cells in the spleen [66]. In another mouse model of triple-negative breast cancer (TNBC), tumor growth was significantly inhibited in the LAG-3 and PD-1 double-blocking mice, and the final tumor volume or weight was also significantly smaller in this group than in the PD-1 or LAG-3 single-blocking group (*P* < 0.05) and PBS control group (*P* < 0.001) after 28 days of treatment and observation [67].

In the CLL mouse model, dual anti-PD-1/LAG-3 therapy reduced the percentage and number of CLL cells in both the blood and spleen, thus effectively reducing the tumor burden in CLL-infected animals, which represented an effective treatment for restoring a functional antitumor immune response. In addition, single anti-PD-1, single anti-LAG-3, single anti-KLRG1 (killer cell lectin-like receptor subfamily G member 1 antibody), and double anti-PD-1/KLRG1 resulted in little or no improvements in CLL progression [68]. Furthermore, Harris-Bookman et al. evaluated the efficacy of combination therapy of anti-LAG-3 (C9B7W, IgG1) and anti-PD-1 monoclonal antibodies in glioblastoma. They found that when compared to no treatment group mice, the combination therapy significantly affected survival (*P* ═ 0.03); moreover, there was a clinical trial involving the combination of anti-LAG-3 and anti-PD-1 in the treatment of glioblastoma (NCT02658981) [69].

US2018326054 described six bispecific antibodies against PD-1/LAG-3 (and their application in the treatment of pancreatic cancer) that were internalized by CD4+ T cells to enhance effector function (involving the release of granzyme B and INF). They found that bispecific antibody therapy against mice inoculated with pancreatic cancer cells resulted in tumor inhibition [70]. Mice with prostate cancer that were immunized with DNA vaccines were treated with either αPD-1, αLAG-3, αPD-1/αLAG-3, or IgG control, and all of the vaccine combinations slowed tumor growth when compared to vaccines with IgG; however, the combination of αPD-1/αLAG-3 with the vaccine resulted in a significant reduction in cancer growth when compared to the administration of either antibody alone [71].

**Figure 2.**
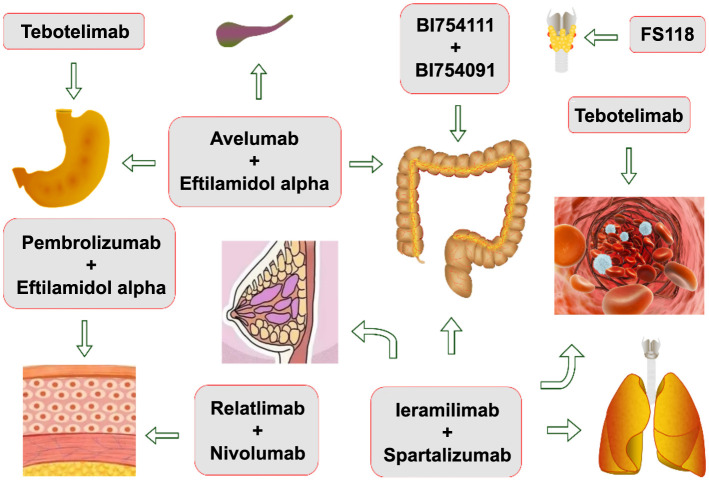
**Clinical evidence of immune checkpoint inhibitors (ICIs).** ICIs were studied in many clinical settings. Tebotelimab is used in gastric cancer and other solid and hematological malignancies. Avelumab and eftilagimod alpha are used in gastric cancer, gallbladder cancer and colon cancer. Pembrolizumab plus eftilagimod alpha or nivolumab plus relatlimab are used in melanoma. BI754091 and BI754111 are used in colon cancer. FS118 is used in anaplastic thyroid cancer. Spartalizumab and ieramilimab are used in breast cancer, non-small cell lung cancer, colorectal cancer, cutaneous melanoma, and metastatic renal cell cancer.

## Clinical evidence

### Melanoma

In a phase 2–3 trial, researchers evaluated relatlimab (anti-LAG-3) and nivolumab (anti-PD-1) as a fixed-dose combination in patients with previously untreated metastatic or unresectable melanoma. The median progression-free survival with relatlimab–nivolumab was longer than that with monotherapy, whereas treatment-related AEs (TRAEs) occurred less frequently with combination therapy [72]. Moreover, 30 patients with resectable clinical stage III melanoma were also treated with neoadjuvant therapy and nivolumab + relatlimab and achieved a high pathologic complete response with a favorable toxicity profile (NCT02519322) [73]. And a phase I/IIa, open-label RELATIVITY-020 trial part D assessed the efficacy and safety of nivolumab and relatlimab in advanced melanoma, including five hundred eighteen patients (D1 ═ 354; D2 ═ 164). The median PFS was 2.1 months (95% CI, 1.9–3.5) in D1 and 3.2 months (95% CI, 1.9–3.6) in D2, and the grade 3–4 TRAE incidence was 15.0% in D1 and 12.8% in D2, which indicates nivolumab and relatlimab had a manageable safety profile and demonstrated durable clinical activity in these patients [74].

In another study, fianlimab (REGN3767, anti-LAG-3) and cemiplimab (anti-PD-1) were assessed in patients with melanoma and showed an acceptable safety profile and some clinical activity (NCT03005782) [75].

A previous study that was divided into Part A (dose escalation) and Part B (extension) evaluated the safety, tolerability, pharmacokinetics, and pharmacodynamics of the combination of eftilamidol alpha (efti, anti-LAG-3) and pembrolizumab (anti-PD-1) in patients with melanoma, and the overall response rate (ORR) was 33% in patients in Part A and 50% in patients in Part B. It was concluded that the combination of efti and pembrolizumab was well tolerated and had good antitumor activity [76]. The majority of patients (83%) treated with efti and pembrolizumab had visceral disease, whereas none of the severe AEs was related to the study treatment (NCT02676869) [77].

In addition, on March 18, 2022, the FDA approved nivolumab and relatlimab-rmbw (Opdualag, Bristol-Myers Squibb Company) for adults and pediatric patients 12 years of age or older with unresectable or metastatic melanoma [78].

### Digestive system cancers and pleural mesothelioma

In patients with unresectable metastatic/locally advanced gastroesophageal junction adenocarcinoma (GEA), the simultaneous targeting of HER2 and PD-1 (margetuximab + retifanlimab) or HER2 and PD-1/LAG-3 (margetuximab + tebotelelimab) resulted in the opportunity to enhance the antitumor response compared to treatment with either agent alone, and currently available data for the coadministration of margetuximab with either retifanlimab or tebotelimab suggested a well-tolerated potential for synergistic antitumor activity, thus supporting the mahogany assay in patients with GEA [79].

In a phase I trial (NCT03156114) that evaluated the combination of BI754111 (anti-LAG-3) and BI754091 (anti-PD-1) in patients with microsatellite stable metastatic colorectal cancer (MSS mCRC), 40 patients with MSS mCRC received combination therapy, three patients had confirmed progressive disease (PD), 11 patients had stable disease (SD), and 5 patients (12.5%) had AEs leading to discontinuation of treatment [80]. In another study, 6 of 8 patients were treated with avelumab and IMP321 for different tumor indications (gastric cancer, gallbladder cancer, colon cancer, and pleural mesothelioma), and treatment with 800-mg avelumab in combination with 6-mg IMP321 was safe and well tolerated [81].

**Table 1:** Results of major preclinical studies of PD-1/LAG-3 inhibitors in vivo

**Tumor type**	**Antibodies**	**Animal model**	**Immune response**	**Results/Conclusions**	**Ref.**
Melanoma	Anti-PD-L1 (10F.9G2), anti-LAG-3 (C9B7W)	Tyrp1B-wRAG-/-Foxp3-DTR TRP-1–specific CD4+ TCR transgenic mice	Tumor-specific Treg-mediated suppression and chronic exhaustion could be overcome with combination treatment	Simultaneous blockade of PD-L1 and LAG-3 in vivo treated recurring tumors	[59]
Mesothelioma	Anti-PD-L1 (10F.92G), anti-LAG-3 (C9B7W)	AB1-HA BALB/cJ mesothelioma mice	Increased secretion of IFNγ, granzyme B and L-2	Survival benefit at the Kaplan–Meier curve	[60]
MC38 cancer, Sa1N fibrosarcoma, B16 tumors	Anti-PD-1 (4H2), anti-mouse LAG-3 (C9B7W)	Lag3^-/-^, Pdcd1^-/-^ and Lag3^-/-^ Pdcd1^-/-^ mice	Higher percentage of IFNγ+ CD8p+ T cells were found, antitumor immunity was enhanced in MC38 cancer and Sa1N fibrosarcoma, but was not effective against established B16 tumors	Mice survived for long time periods	[92]
Colon cancer	mLAG-3/PDL1 mAb^2^	C57BL/6 mice or Balb/c mice	Enhancing the antitumor immune response	Inhibited tumor growth in vivo	[62]
Colon cancer	CB213	Transgenic hPD1/ hLAG3 HuGEMM mice	Significant levels of tumor-infiltrating lymphocytes were observed	64% tumor growth inhibition (TGI)	[63]
Colon cancer	REGN3767, cemiplimab	Human PD-1xLAG-3 knock-in mice	The secretion of proinflammatory cytokines by tumor-specific T cells was enhanced	Showed increased efficacy in a mouse tumor model	[47]
Non-small cell lung cancer	TSR-042, TSR-033	HuNOG-EXL mice	Combination treatment increased T cell proliferation, IFNγ production, and elicited durable immunological memory	Impeded tumor growth to a greater extent compared to either monotherapy	[66]
Breast cancer	NE-purified anti-mouse PD-1 antibodies, purified NA/LE anti-mouse LAG-3 antibodies	Female BALB/c mice	Dual blockade of LAG-3 and PD-1 could induce a stronger antitumor effect	Significantly inhibited tumor growth in mice	[67]
Glioblastoma	Anti-murine PD-1 monoclonal antibody, anti-LAG-3 (C9B7W)	Female C57BL/6J mice	The percentage of CD8 or CD4 IFNγ producing cells (T effector cells) was not significantly different across groups although the combination trended toward higher percentage of effector cells	Inhibition of LAG-3 improved survival in a preclinical glioblastoma model and considerably improved the efficacy of anti-PD-1 treatment	[69]

LAG-3: Lymphocyte activation gene 3; PD-1: Programmed cell death protein 1; INFγ: Interferon gamma; Treg: Regulatory T cells.

### Other tumors

PD-1 and LAG-3 have also been researched in other tumors. Five patients with TNBC participated in phase I/II clinical trial of anti-LAG3 LAG525 in combination with or without anti-PD-1 spartalizumab in advanced malignancies. Two of these patients showed objective responses with a tendency to convert an immune-cold into an immunoactive biomarker on tumor biopsies, and another early targeting strategy for LAG-3 involved the bispecific monoclonal antibody tebotelimab (MGD013), which cotargeted LAG-3 and PD-1 [82–84]. In a study of RCC, the results indicated that PD-1/LAG-3 (rather than PD-1/TIM-3 blockade) improved the immune function of stimulated RCC TILs (*P* ═ 0.0302, Fisher’s exact test) [85].

In another previous study, the researchers presented a case of a patient with anaplastic thyroid cancer (ATC) who progressed under multiple treatment regimens with a sustained and durable response to FS118 (bispecific anti-PD-L1 and anti-LAG-3); the treatment was consistently well tolerated, and the patient had persistent disease and clinical benefit [86]. In phase I/II, multicenter study (NCT02460224), more than 200 patients with several tumor types (NSCLC, colorectal cancer, cutaneous melanoma, metastatic RCC.) were treated with ieramilimab (LAG525, anti-LAG-3) in combination with or without spartalizumab (PDR001, anti-PD-1), and this combined application was well tolerated as monotherapy with obvious clinical benefits [87, 88].

**Table 2:** Results of major clinical studies of PD-1/LAG-3 inhibitors for tumor treatment

**Tumor type**	**Drug(s)**	**Trial phase**	***N***	**Main objectives**	**Results/Conclusions**	**Clinical trial**	**Ref.**
Melanoma	Nivolumab+relatlimab	Phase II/III	714	Median PFS	PFS: 10.1 months (95% CI, 6.4–15.7)	NCT03470922	[72]
Melanoma	Nivolumab+relatlimab	Phase II	53	pCR rate	pCR rate: 59%	NCT02519322	[73]
Melanoma	Pembrolizumab+eftilagimod alpha	Phase I	24	Safety, tolerability, PK and PD	ORR of part A: 33%; ORR of part B: 50%	NCT02676869	[76]
Gastric/gastroesophageal junction adenocarcinoma	Tebotelimab	Phase II/III	82	ORR, overall survival and safety/tolerability	There was potential synergic antitumor activity with good tolerability	NCT04082364	[79]
Colorectal cancer	BI754091+BI754111	Phase I	172	Evaluating the combination of BI 754111 and BI 754091 in patients with advanced solid tumors	CR: 0%; PR: 7.5%	NCT03156114	[80]
Thyroid cancer	FS118	Phase I/II	80	Case report	FS118 afforded patient a sustained partial response with excellent tolerability	NCT03440437	[86]
Solid tumors	Avelumab+eftilagimod alpha	Phase I	45	Feasibility and safety	Combination treatment was safe and well tolerated	NCT03252938	[81]
Solid or hematologic malignances	Spartalizumab+ieramilimab	Phase II	76	Preliminary efficacy	Combination treatment showed promising activity	NCT03365791	[91]
Advanced malignancies	Spartalizumab+ieramilimab	Phase I/II	490	Assessing the maximum tolerated dose (MTD) or recommended phase II dose	Combination treatment had modest antitumor activity	NCT02460224	[87]
Advanced malignancies	Cemiplimab+REGN3767	Phase I	333	Initial safety, PK, and efficacy from the dose escalation study of combination treatment	The safety profile of REGN3767 ± cemiplimab was generally tolerable; PK was linear	NCT03005782	[90]

N: Number of participants (actual/estimated enrollment) in the clinical study; PFS: Progression-free survival; pCR: Pathologic complete response; ORR: Overall response rate; CR: Complete response; PR: Partial response; PK: Pharmacokinetics; PD: Pharmacodynamics.

In a previous study, researchers studied the safety, tolerability, dose-limiting toxicity, maximum tolerated dose (MTD), and antitumor activity of MGD013 (which is an experimental bispecific molecule designed to bind PD-1 and LAG-3) in patients with advanced solid and hematological malignancies, and MGD013 synergistically blocked PD-1 and LAG-3 with acceptable safety and preliminary evidence of antitumor activity [84]. In another study, 17 diffuse large B-cell lymphoma (DLBCL) patients also received MGD013, and serum IFN-γ was significantly increased > 140-fold above baseline, as well as associated lytic markers (i.e., perforin and granzyme B) [89]. When 42 patients with advanced malignancies were given REGN3767 and cemiplimab, the safety profile was generally tolerable, and early efficacy signals were detected [90]. In a phase II study that was conducted in patients with solid or hematologic malignances, patients received spartalizumab + LAG525, and the combined therapy showed promising activity in neuroendocrine tumors, SCLC, and DLBCL that met the expansion criteria (NCT03365791) [91].

## Discussion

The blockage of immune checkpoints to treat cancer has greatly improved the prognosis of cancer patients. The use of ICIs, especially regarding their combination therapy, has been extensively studied in clinical (Figure 2) and preclinical settings with encouraging results in the cancers described above (Tables 1 and 2). Although dual blockade has shown promising therapeutic effects in many tumor models, such as melanoma, ATC, and others [72, 76, 86–88], there are still issues that need to be addressed, such as the low response rate of some tumors to this therapy, which is regarded as the least immunogenic [92]. In addition, the clinical benefit of the combination came at the expense of an increased incidence of autoimmune toxicity [93]. AEs are very common, such as fatigue, nausea, gastrointestinal disorders, and skin disorders, although severe AEs are rare [87–89]. Furthermore, the types and doses of the tested drugs were limited, and several combinations of relatlimab, nivolumab, efti, pembrolizumab, ieramilimab, spartalizumab, cemiplimab, and tebotelimab were assessed with some doses in clinical applications [72, 76–78, 81, 87, 88, 91]. Other potential combinations may need to be evaluated. Finally, although the overall number of studies is large, there have been few large-scale and systematic studies; thus, more research is required to support this combination therapy. Overall, the combination of PD-1 and LAG-3 blockers is very promising, but more extensive and in-depth research is needed to determine the best drug type and dose combination, improve the response rate of patients, and reduce TRAEs.

Although the combination therapy is promising, there are still many challenges. Combination therapy is more effective than monotherapy, but the ORR is still not high. The toxicity of combination therapy is not significantly increased compared to monotherapy, but AEs remain a thorny problem. In subsequent studies, the selection of appropriate combination drugs and dosages in different tumors is also a major challenge. The addition of anti-LAG-3 alleviates anti-PD-1 resistance to some extent, but resistance still exists. In recent years, some scholars have put forward new ideas. Patients who are refractory to anti-PD-1 inhibition or anti-CTLA-4 antibody have shown clinically meaningful activity when given anti-LAG-3 antibodies, which suggests nonoverlapping mechanisms of antitumor immune activity. It is significant to know whether anti-CTLA-4 also has efficacy in patients who had disease progression while receiving anti-PD-1 plus anti-LAG-3. Unfortunately, the early data suggest that tumors are unlikely to respond to CTLA-4-targeted therapy when they are refractory to anti-PD-1 inhibition and anti-LAG-3 antibody, more study is still needed [94].

In addition, although there have been many preclinical or clinical studies of immunotherapy and combination therapy of immunotherapy, only melanoma and NSCLC have made some progress, and the effect of other tumors is still controversial, especially in sarcomas. And the current clinical drugs are mainly monotherapy, the FDA has approved the combination of nivolumab and relatlimab for melanoma, but further research is still needed for other tumors.

At present, there are still differences in the view of clinical application. Although some patients have been relieved, many patients still cannot benefit from it. At the same time, clinical application faces the challenges of serious AEs, drug resistance, and high medical costs, and predictive biomarkers of response to immunotherapy including PD-L1 require further research. Combinations require large cohort studies to address significant clinical validations. These challenges are the knowledge gaps of combinations of immunotherapy, the treatment strategy may be moving in the direction of solving these problems. Fortunately, there are a number of ongoing clinical trials evaluating the efficacy and safety of other combined PD-1 and LAG-3 antitumor therapies, these problems are expected to be resolved within five years.

Our work also has some limitations. For example, we did not explore in detail the molecular mechanisms of the combination and the mechanisms of drug resistance, the list of articles is not comprehensive on the combination application, and the studies covered in the article may also have a publication bias.

## Conclusion

Ultimately, the use of PD-1 and LAG-3 blockers has notably improved response and survival rates for numerous types of cancer. Nevertheless, frequent AEs were observed. Additional research may be necessary to enhance patient response rates, minimize TRAEs, and determine the most effective drug type and dosage combination.
